# Advancing Immunotherapeutic Vaccine Strategies Against Pulmonary Tuberculosis

**DOI:** 10.3389/fimmu.2020.557809

**Published:** 2020-09-09

**Authors:** Sam Afkhami, Anne Drumond Villela, Michael R. D’Agostino, Mangalakumari Jeyanathan, Amy Gillgrass, Zhou Xing

**Affiliations:** ^1^McMaster Immunology Research Center, McMaster University, Hamilton, ON, Canada; ^2^Department of Pathology and Molecular Medicine, McMaster University, Hamilton, ON, Canada; ^3^Michael G. DeGroote Institute for Infectious Disease Research, McMaster University, Hamilton, ON, Canada

**Keywords:** tuberculosis, therapeutic vaccine, chemotherapy, immunotherapy, respiratory mucosa, mycobacterial life cycle

## Abstract

Chemotherapeutic intervention remains the primary strategy in treating and controlling tuberculosis (TB). However, a complex interplay between therapeutic and patient-related factors leads to poor treatment adherence. This in turn continues to give rise to unacceptably high rates of disease relapse and the growing emergence of drug-resistant forms of TB. As such, there is considerable interest in strategies that simultaneously improve treatment outcome and shorten chemotherapy duration. Therapeutic vaccines represent one such approach which aims to accomplish this through boosting and/or priming novel anti-TB immune responses to accelerate disease resolution, shorten treatment duration, and enhance treatment success rates. Numerous therapeutic vaccine candidates are currently undergoing pre-clinical and clinical assessment, showing varying degrees of efficacy. By dissecting the underlying mechanisms/correlates of their successes and/or shortcomings, strategies can be identified to improve existing and future vaccine candidates. This mini-review will discuss the current understanding of therapeutic TB vaccine candidates, and discuss major strategies that can be implemented in advancing their development.

## Introduction

In 2014, the World Health Organization (WHO) supported a post-2015 END TB Resolution that aimed to curb 90% of both cases and deaths associated with tuberculosis (TB) by 2035 (1). Although measurable progress toward these goals has been made to-date, recent reports show that many interim goals originally set for 2020 will not be met globally (2). A major proportion of these shortcomings stem from current anti-TB chemotherapy regimens including fragmented patient adherence, treatment failure, and emergence of multi- and extensively drug resistant disease (3). Novel strategies to be administered in adjunct with conventional chemotherapy, known as Host-Directed Therapies (HDT), are designed to combat such shortcomings by improving patient adherence, improving cure rates, and preventing disease relapse (4, 5). In this mini-review, we focus on therapeutic vaccination strategies as HDTs for TB. We discuss current requirements for therapeutic vaccines in accordance with WHO standards, their mechanisms of action, detail the current pipeline, and expand on strategies to improve therapeutic vaccines.

## Current TB Chemotherapy Landscape and Its Shortcomings

Chemotherapy for drug-susceptible TB requires two months of continuous treatment with a cocktail of rifampicin, isoniazid, ethambutol, and pyrazinamide followed by four months of rifampicin and isoniazid. Although this regimen has remained largely unchanged for the last 35 years, numerous healthcare policies such as Direct Observed Treatment Short course have allowed for cure rates as high as 85% (2, 6). This, however, starkly contrasts the low success rate (56%) for drug-resistant disease, for which treatment can last upwards of 24 months (2). Although there are numerous factors that contribute to such low rates, many arise from, and are further exacerbated by poor patient adherence due to the duration of treatment required to successfully treat disease.

It is important to note that novel drugs and drug regimens progressing through the clinical pipeline continue to substantially improve TB treatment outcomes. Novel chemotherapeutics such as bedaquiline and pretomanid have allowed for regimens as short as nine months to be effective in curing drug-resistant forms of TB (7). However, despite such continued improvements, patient adherence remains poor. Chemotherapy durations remain longwinded and costly, and novel antibiotics pose the threat of off-target toxicities such as peripheral neuropathy and cardiac manifestations (8). Non-compliance additionally drives drug resistance which has alarmingly already been documented for bedaquiline (9). These, alongside other socioeconomic issues, are entangled with the longevity of treatment and therefore remain a major roadblock toward the success of chemotherapy against TB.

The development of novel immunotherapeutic strategies that synergize with antibiotics to further shorten the duration of chemotherapy required to successfully treat disease is an alluring platform for improving patient compliance and treatment success.

## Rationale Behind Prolonged Multi-Drug Regimens

Logically, multiple drugs are required to treat TB as to avoid the selection of resistant mycobacterial subpopulations that arise from single-drug monotherapy (defined as genetic tolerance) (10). Therefore, individuals with a higher bacillary burden are statistically more likely to carry intrinsically resistant mutants. In stark contrast, the rationale behind the length of therapy is perhaps less intuitive and requires scrutinization of the host-pathogen interface.

Airway macrophages comprise the major niche for *Mycobacterium tuberculosis* (*M.tb*) infection and outgrowth (11, 12). Infected macrophages deploy numerous mechanisms to eliminate *M.tb* which include phagolysosome fusion, upregulation of antimicrobial peptides, and autophagy (13–15). Eventual priming and recruitment of adaptive Th1 immunity further enhances mycobacterial control in part by promoting innate-mediated killing (16). Unfortunately, innate and adaptive immune responses together are often unable to fully eliminate the pathogen. This necessitates addition of chemotherapeutic interventions to further enhance mycobacterial clearance through inhibition of cell wall synthesis (isoniazid and pyrazinamide), and transcription (rifampicin) (10).

However, *M.tb* is fully capable of adapting to both the immunological and pharmacological pressures placed upon it. Examples of such bacterial countermeasures include inhibiting phagosome acidification, delaying adaptive responses, and upregulation of drug efflux pumps (15–18). In addition, immunological and chemotherapeutic stresses also cause *M.tb* to undergo transcriptomic and metabolic shifts leading to a state of dormancy. In this non-replicative state, the antigenic profile of *M.tb* shifts toward expression of stress/dormancy associated genes (19–25). Collectively, this not only further circumvents adaptive immune responses established against the replication antigens, but it also thwarts the efficacy of such therapies (defined as phenotypic tolerance) since the majority of TB chemotherapeutics only target actively replicating bacilli. These phenotypically tolerant, non-replicating mycobacteria are a major and under-investigated subpopulation responsible for disease relapse and the need for prolonged antibiotic therapy (26). As such, there has been a growing interest in strategies which can address such problems. Host-directed therapies represent a promising therapeutic category designed to accomplish this goal.

## Improving Treatment Outcome With Host-Directed Therapies

Host-directed therapies are administered in tandem with conventional chemotherapy to improve treatment success and reduce disease relapse. Depending on the strategy, HDTs accomplish this by (1) augmenting the host anti-TB immune response, (2) limiting lung pathology, and/or (3) enhancing mycobacterial sensitivity to chemotherapy. Numerous drug-based HDTs are currently under pre-clinical and clinical investigation. For example, the antihyperglycemic agent metformin has shown promise as it enhances macrophage intracellular mycobacterial killing by increasing phagolysosome fusion and production of reactive oxygen species (27, 28). Drug-based HDTs have been reviewed extensively elsewhere and will not be the focus of this review (4, 5, 29).

## Vaccines as Host-Directed Therapies Against TB

Tuberculosis vaccination strategies encompass those administered prophylactically (to prevent infection), therapeutically (to improve treatment of active disease), and post-exposure (to prevent re-infection/re-activation) (30). Therapeutic vaccines continue to gain traction as leading candidates for TB HDTs.

**FIGURE 1:**
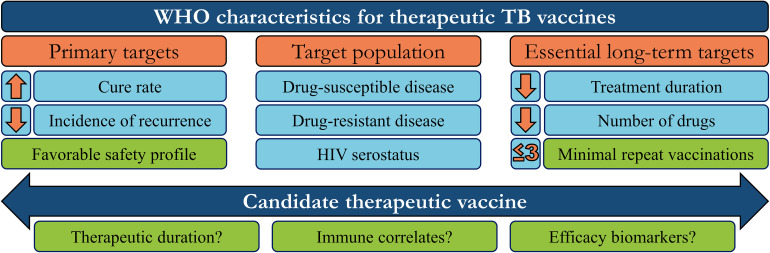
WHO target characteristics for therapeutic tuberculosis vaccines. In order to guide research and development, the WHO has set aspirational targets/characteristics for candidate therapeutic TB vaccines. These include three major areas to address (orange boxes): Vaccine targets that determine efficacy, the target population, and the subsequent long-term impact on the chemotherapy regimen. Candidates which meet some/all of these targets must also be safe, efficacious in a minimum number of repeated doses, and investigated to define their mechanism-of-action or potential efficacy biomarkers, to further refine/guide future vaccination strategies (green boxes).

**FIGURE 2:**
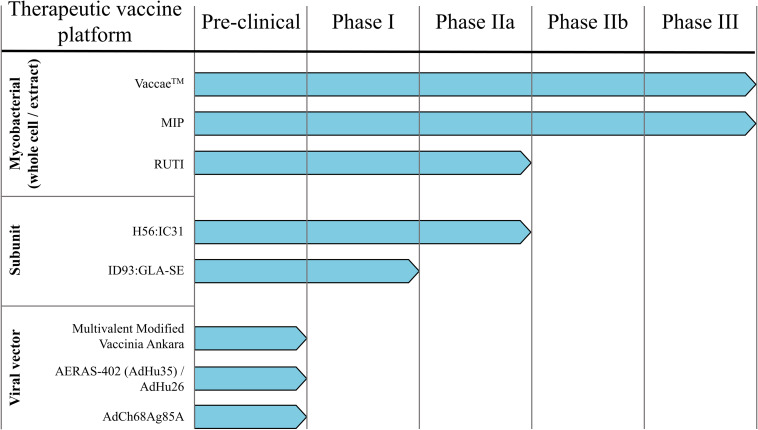
Therapeutic TB vaccine pipeline. Numerous TB vaccine candidates under pre-clinical and/or clinical testing for their prophylactic efficacy are also evaluated as immunotherapies.

Therapeutic vaccines are administered in adjunct (at the start or during) with conventional chemotherapy to accelerate treatment, shorten chemotherapy duration, and improve treatment completion rates. The WHO has generated a detailed list of parameters necessary for an ideal therapeutic vaccine candidate, of which the major targets/goals are presented in Figure 1 (31). Therapeutic TB vaccines aspire to accomplish these goals by boosting host anti-TB immunity, priming novel immune responses, and modulating the host inflammatory response. The following section briefly outlines the most recent advancements in emerging therapeutic TB vaccines, as identified by the TuBerculosis Vaccine Initiative (TBVI)^1^ and the 2018 WHO Global TB Report (Figure 2).

## Mycobacterial-Based Therapeutic TB Vaccines

### Vaccae^TM^

Vaccae^TM^ consists of a heat-killed *Mycobacterium vaccae* (*M vaccae*) variant (32). This non-tuberculous mycobacterium is currently the most advanced therapeutic TB vaccine candidate and is approved for administration alongside standard chemotherapy in patients with active TB in China (33, 34). Although clinical data pertaining to Vaccae^TM^ efficacy is conflicting (with certain meta-analyses indicating little-to-no improvement), recent studies have highlighted its therapeutic efficacy (35, 36). In particular, such studies have highlighted the ability of orally administered Vaccae^TM^ to accelerate negative sputum smear conversion. A large-scale phase III trial with 10,000 recruited participants is ongoing with interim results showing high retention rates (clinicaltrials.gov, NCT01979900). However immunological data pertaining to Vaccae^TM^ mechanism of action is limited. Pre-clinical and clinical immunological studies have suggested that induction of robust Th1 (and conversely skewing away from Th2) immune responses, alongside induction of cytotoxic CD8 T lymphocytes are correlatives of efficacy (37).

### Mycobacterium indicus pranii

*Mycobacterium indicus pranii* (MIP) is a non-pathogenic mycobacterium whose heat-killed variant has been successfully used as an immunotherapeutic for leprosy (38, 39). Characterization of the antigenic profile of MIP has shown broad antigenic congruency with *M.tb*, warranting further investigation toward its potential as a therapeutic vaccine. A recently published phase II trial where MIP was intradermally administered in adjunct to chemotherapy showed success in enhancing bacterial clearance, and improved lung pathology in patients with bi-lateral, drug-resistant disease (40). Pre-clinical studies in numerous animal models (including hyper-susceptible guinea pigs) have correlated the therapeutic efficacy of MIP to its ability to drive robust Th1-skewed immunity (41–43). Interestingly, these studies also compared the contribution of immunization route to efficacy, showing that respiratory mucosal vaccination was immunologically superior to the parenteral route.

### RUTI^®^

In contrast to whole, heat-killed mycobacteria, RUTI^®^ is composed of liposomes containing detoxified fragments of *M.tb* grown under hypoxic/stress conditions (44, 45). The growth of virulent *M.tb* under these conditions drives expression of an array of stress and latency associated antigens, thereby expanding the antigenic breadth of the RUTI^®^ formulation, theoretically allowing for adaptive immune responses to target latent bacilli (46). The added advantage of this strategy is targeting the latent bacillary population which, as stated previously, is a major contributor to disease relapse. RUTI^®^ is currently being investigated as a subcutaneously administered post-exposure vaccine in individuals with drug-resistant disease who have completed chemotherapy (45, 47). As such, RUTI^®^ remains to be tested therapeutically in patients with active disease, potentially due to issues related to the Koch phenomenon (45).

## Recombinant-Based Therapeutic TB Vaccines

### H56:IC31

H56:IC31 is a recombinant fusion protein of three *M.tb* antigens: Ag85B, ESAT-6, and Rv2660c combined in a stabilizing agent containing a TLR9 agonist as an adjuvant (48). By expressing replication and pathogenicity-based antigens (Ag85B, and ESAT-6, respectively) and latency-associated antigens (Rv2660c), H56:IC31 is designed to drive multi-functional Th1 immunity against both actively replicating and dormant bacilli, and has been shown to prevent *M.tb* reactivation in macaques following parenteral delivery (48, 49). H56:IC31 has undergone extensive clinical testing as a prophylactic vaccine in phase 1 and 2 trials showing a favorable safety and immunogenicity profile in IGRA− and IGRA+ individuals (50). In line with its success and clinical advancement as a prophylactic vaccine, H56:IC31 is also being investigated as an intramuscularly administeredtherapeutic for TB. This includes a phase 1 combinatorial HDT regimen with a Cyclooxygenase-2 inhibitor etoricoxib (clinicaltrials.gov, NCT02503839), and a phase II efficacy trial in South Africa addressing prevention of disease reoccurrence (clinicaltrials.gov, NCT03512249).

### ID93/GLA-SE

ID93/GLA-SE is a recombinant vaccine expressing three virulence (Rv2608, Rv3619, and Rv3620) and one latency-associated (Rv1913) antigen in combination with a synthetic TLR4 agonist in an oil-in-water emulsion (51, 52). Similar to the other vaccine candidates described in this review, ID93/GLA-SE has been extensively characterized in pre-clinical animal models. Such studies have shown that ID93/GLA-SE drives robust and sustained anti-TB specific CD4 and CD8 T cell responses following parenteral delivery, correlating with significantly improved prophylactic protection against pulmonary TB (53). Therapeutic vaccination by the intramuscular route with ID93/GLA-SE in non-human primate models significantly reduced pulmonary mycobacterial burden, pathology, and improved survival (53). Thus far, a phase I trial has shown that ID93/GLA-SE is safe and immunogenic in QuantiFERON positive individuals, thereby supporting its further assessment as an immunotherapeutic (54).

## Viral-Vectored Therapeutic TB Vaccines

### Modified Vaccinia Ankara

Modified Vaccinia Ankara (MVA)-based TB vaccines have a checkered history with a phase IIb trial of MVA85A failing to enhance prophylactic immunity following parenteral immunization in BCG-vaccinated infants (55). Regardless, MVA-vectors remain widely utilized as TB vaccines given their remarkable ability to accommodate large transgene inserts and drive long-lived Th1 immunity (56). A recently developed MVA-based vaccine expressing 10 *M.tb* antigens has been assessed for its therapeutic efficacy in a murine model. Interestingly, this vaccine provided minimal reduction of bacterial burden within the lungs and a modest reduction in relapse (57). This was observed following parenteral, but not respiratory mucosal immunization. Additionally, immunological studies revealed that immune responses against some antigens were not induced. These observations highlight certain key considerations for vaccine design which will be addressed further in this review.

### Adenovirus

Adenoviruses represent the most widely used viral vector platform for vaccine design (58). Human adenoviruses, in particular human adenovirus serotype 5 (AdHu5), have been extensively tested as a prophylactic vaccine platform delivered both parenterally and via the respiratory mucosal route (58–62). Unfortunately, in some instances the high seroprevalence of antibodies against this common respiratory pathogen has limited its efficacy. This has prompted the development of platforms founded in serotypes exotic to humans (63).

Non-human adenoviruses, such as chimpanzee adenoviruses have gained traction as vaccine vector candidates given their robust immunogenicity and low seroprevalence (63).

We previously characterized the prophylactic potential of a chimpanzee adenoviral-vectored vaccine expressing *M.tb* Ag85A (AdCh68Ag85A) (64). Our study showcased the superior immunogenicity and efficacy of this vector following respiratory mucosal administration in comparison to its AdHu5 counterpart. Additionally, utilizing a clinically relevant murine model of chemotherapy treated active TB disease, our group has shown that respiratory mucosal therapeutic immunization with AdCh68Ag85A accelerated bacterial clearance, limited lung pathology, and limited disease relapse following pre-mature chemotherapy cessation (65).

## Advancing Therapeutic TB Vaccine Design

**FIGURE 3:**
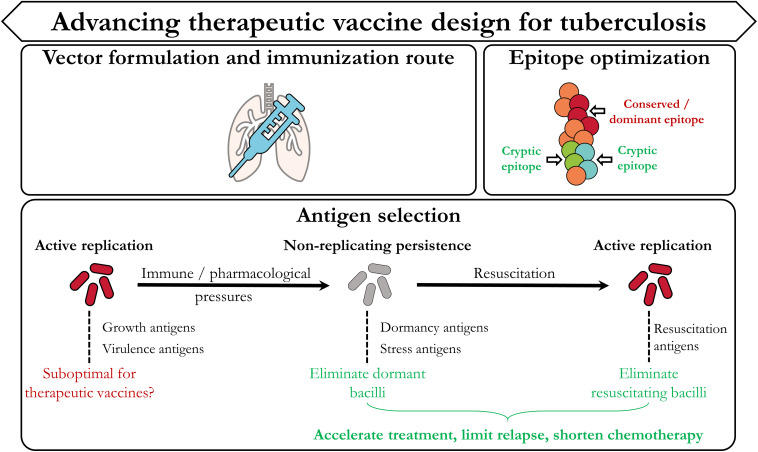
Designing improved therapeutic TB vaccination strategies. In accordance with Figure 1, the current TB vaccine landscape has provided invaluable evidence which can be used to improve therapeutic vaccination strategies. These include the type of vaccine vector, the route of immunization, the selection and design of the epitope(s)/antigen(s), and further focus on identifying targets from the different stages of the mycobacterial life cycle.

Although definite protective immune correlates for TB remain elusive, insights from ongoing pre-clinical and clinical trials provide invaluable information to steer development of efficacious therapeutic TB vaccination strategies. Such insights stem from both the immunological as well as the mycobacterial areas of research. In this section we highlight three categories (Figure 3) which we believe are most important in advancing therapeutic TB vaccine design: Vector formulation and immunization route, antigen/epitope optimization, and antigen selection dictated by the mycobacterial life cycle.

### Vector Choice

Factors such as feasibility, safety, and immunogenicity are largely dictated by vector choice. As such, this represents a major consideration when developing novel TB therapeutic vaccine strategies. Whole organism-based vaccines (Vaccae^TM^ and PIM) are advantageous given their antigenic breadth. These vectors however may not be amenable for delivery via the respiratory mucosal route in humans due to potential safety considerations. Additionally, utilization of whole mycobacteria/mycobacterial fragments can potentially skew and/or mask immunological responses against less immunogenic, yet protective antigens (see below). As such, utilization of recombinant molecular biology techniques allows for construction of recombinant vaccines that focus immune responses against well-recognized protective antigens. This is the case for recombinant protein and viral-vectored vaccination strategies (58, 66).

In addition, through careful adjuvant selection, antigen-specific immunity can be polarized toward certain immune profiles. Use of TLR agonists (such as TLR9 in H56:IC31and TLR4 in ID93:GLA-SE) enhance both the cytokine functionality and longevity of Th1 immune responses which are critical for anti-TB immunity (67). Identification of adjuvants capable of inducing cytotoxic CD8 T cell responses can be challenging however and may represent a roadblock in developing optimal therapeutic TB vaccines. We have recently shown that antigen-specific CD8 T cells are the major cell type involved in the therapeutic efficacy of AdCh68Ag85A, thereby providing supporting evidence of their importance in anti-TB immunity (65).

Viral vectors, particularly adenoviruses, remain the most widely utilized platforms for vaccine design given their genetic malleability, safety, and amenability for respiratory mucosal administration (58). However as echoed by us and others, viral serotype selection is a major consideration in the downstream immunogenicity and efficacy of a vaccine candidate. For example, human adenovirus serotype 35 (AdHu35) has been used in place of AdHu5 given its low seroprevalence, which allows it to circumvent anti-vector immunity (68). Thus, this vector has been extensively tested as a putative prophylactic TB vaccine candidate (AERAS-402) (69). Unfortunately, recent studies have shown that parenteral administration of this vaccine did not significantly induce antigen-specific immunity (70, 71). In line with this observation, it is well documented that AdHu35 induces robust type 1 interferon responses, leading to downstream loss of vector transgene expression, which may negatively impact T cell immunity (72). Alongside the choice of immunization route (explained below), this also may explain why AERAS-402 failed to confer efficacy when administered therapeutically (73).

Progress in the development of other novel vaccine platforms provide new opportunities in further advancing therapeutic TB vaccine development. For example, nanoparticle-based vaccines have shown tremendous progress against a plethora of infectious diseases (74). Nanoparticles not only act as antigen carriers, but also possess intrinsic immunogenic properties (often not requiring adjuvants) which can be fine-tuned by altering their physiochemical properties (75). Recent studies have shown that nanoparticle-based TB vaccines are not only amenable for respiratory mucosal delivery, but can drive robust Th1-skewed immune responses which provide similar-to-grater immunity than BCG alone (76, 77).

### Immunization Route

Compelling evidence suggests that protection against mucosal pathogens such as *M.tb* is heavily reliant on the presence of pathogen-specific immune cells at the primary site of infection (78–80). As seen during natural *M.tb* infection, bacterial control is observed when anti-TB specific T cells appear in the lung (81). Specifically, it is the presence of such immune responses within the airways that is critical in anti-TB immunity. Immunization route largely determines the anatomical location of antigen-specific T cells and therefore, vaccine efficacy (82). Pre-clinical studies show that parenteral immunization with TB vaccines drives robust antigen-specific T cell immune responses. However, such cells are primarily restricted to the periphery, unable to quickly enter the lung interstitium and airway lumen, and largely fail to confer protection against pulmonary TB (83). In stark contrast, respiratory mucosal immunization generates a long-lasting population of tissue-resident polyfunctional T cells that are primed to express homing molecules to allow preferential migration and residence in the airway lumen and lungs (80, 82, 84, 85). These immune cells, located at the portal of infection, are able to rapidly respond and carry out their effector function, providing markedly enhanced protection against pulmonary *M.tb* infection.

### Antigen/Epitope Optimization

Most TB vaccine candidates in development continue to utilize antigens that are widely characterized in *M.tb*-infected individuals and are proven to be immunogenic (e.g., Ag85A, ESAT6, TB10.4). Seminal studies however have shown that *M.tb* infection in humans is characterized by adaptive T cell responses which are skewed toward hyper conserved epitopes (86, 87). Broad conservation of immune responses against such epitopes is a non-prototypic measure of immune evasion which *M.tb* is speculated to have evolved to control and concentrate the host immune response against non-protective antigens. As most vaccine strategies are formulated to include such immunodominant antigens and have been ineffective, it suggests that these candidates may be suboptimal for vaccine-derived protection. Consequently, targeting non-dominant (cryptic) antigens may represent a superior avenue for designing efficacious vaccines (88).

Expanding the repertoire of T cell immunity through immunization with cryptic antigens has been evaluated in TB in pre-clinical models (89, 90). Such studies have shown that T cell responses against cryptic antigens are significantly more functional than those elicited against classical immunodominant antigens and provide enhanced protection. Importantly, T cell responses against such cryptic antigens are longer-lived, and are less prone to exhaustion, making them ideal for long-lasting immunity. Collectively, such observations warrant the inclusion of cryptic antigens in therapeutic vaccination strategies.

### Antigen Selection

Following pulmonary infection, adaptive immune responses are primarily skewed toward replication and virulence-associated antigens. As previously mentioned, immunological and pharmacological stresses proceed to drive *M.tb* into a non-replicating, dormant state. Antigenically, this is associated with a shift from replication-associated antigens to those involved in the stress and the dormancy response. Consequently this allows *M.tb* to evade existing adaptive immune responses that have already been primed against such antigens. Two major conclusions can be inferred from this: Only targeting replication-associated antigens may be suboptimal for therapeutic vaccination platforms, and including dormancy/stress-associated antigens may improve therapeutic vaccine efficacy. A plethora of dormancy antigens are currently under investigation as tantalizing vaccine antigen candidates. As detailed earlier, H56:IC31 expresses the latency antigen Rv2660c and ID93:GLA-SE expresses Rv1813 (48, 53). These represent just two of many potential dormancy candidates (such as those controlled by the DosR Regulon) that warrant further investigation. In addition, it will be critical to assess whether immune responses to such antigens are able to specifically eliminate dormant bacilli. To do so will require implementation and standardization of experimental techniques that delineate between these mycobacterial subpopulations, which have been described previously (91).

In addition, targeting *M.tb* populations resuscitating from dormancy would also be critical in the development of therapeutic vaccines, as to eliminate/minimize reactivation. Mycobacterial resuscitation is a complex process and is regulated in a coordinated transcriptional burst preceding expression of metabolic and growth-related pathways (92). Resuscitation promoting factors (RPFs) represent a major class of antigens involved in this process and may represent promising vaccine antigen targets (92–94).

Evidence supporting further investigation of RPFs as vaccine antigens stems from clinical studies that correlated the long term maintenance of multifunctional adaptive immunity against these antigens in *M.tb*-infected non-progressors (95). This strongly supports the role of such immune responses in controlling reactivation from latency. Although RPFs have been included in numerous pre-clinical prophylactic vaccine candidates, no study to-date has investigated the contribution of RPF-specific immunity in restricting resuscitation (96, 97). Given the significant contribution of resuscitating *M.tb* populations to disease relapse, developing therapeutic vaccines to improve immune surveillance during or post-chemotherapy against such subpopulations would have substantial benefits.

## Concluding Statement

Reaching the END TB set goals for 2035 remains a daunting task which we are unlikely to meet short of a revolution in current treatments. Investigation into therapeutic vaccines has expanded rapidly in the last several years offering novel insights toward vector design, and targeted antigen selection from differing stages of the bacterial lifecycle. While this field lags behind the development of prophylactic strategies, therapeutic vaccines have the potential to enhance treatment success rates, even for difficult-to-treat drug-resistant forms of TB. As this emerging field of TB vaccine development continues to flourish, there are several factors that will be critical to assessing their potential success, and eventual utilization. Namely, to what extent are these vaccines able to shorten the duration of existing chemotherapeutic regimens? How will we evaluate the efficacy of these vaccines under realistic situations such as fragmented or incomplete therapy? Can therapeutic immunization protect against recurrent infection? Intertwined with this are establishing correlates to measure efficacy, and the standardization of complex, pre-clinical models necessary to account for the myriad of moving parts at play. This includes, but is not limited to, dissecting immune responses from vaccines versus those induced from infection, the role of chemotherapy in driving dormancy/escape from immunological pressures, and safety/efficacy in individuals living with HIV.

## Author Contributions

SA, AV, and ZX designed the manuscript. SA and AV wrote the manuscript. ZX approved the manuscript. All authors revised and edited the manuscript.

## Conflict of Interest

The authors declare that the research was conducted in the absence of any commercial or financial relationships that could be construed as a potential conflict of interest.

The reviewer HM declared a past co-authorship with one of the authors ZX to the handling Editor.

## Footnotes

1 https://www.tbvi.eu/
